# Current concepts in meniscal suturing techniques: indications, advantages, and limitations

**DOI:** 10.1530/EOR-2025-0236

**Published:** 2026-07-01

**Authors:** Marta Gabriela Kubisa, Michał Jan Kubisa, Agnieszka Grygiel, Robert Omilian, Przemysław Krakowski

**Affiliations:** ^1^ Departament of Orthopaedics and Traumatology of Carolina Hospital, Warsaw, Poland; ^2^ Departament of Orthopaedics and Traumatology of Medical University of Warsaw, Warsaw, Poland; ^3^ Masovian Center for Neuropsychiatry, Warsaw, Poland

**Keywords:** meniscus repair, inside-out, outside-in, all-inside, knee arthroscopy

## Abstract

- The meniscus is critical for load distribution and joint stability; tears disrupt biomechanics and accelerate cartilage degeneration, prompting a shift from meniscectomy toward preservation and repair.
- We review three core techniques – inside-out, outside-in, and all-inside – with stepwise descriptions, indications, advantages/limitations, devices, and complication profiles. Inside-out offers precise, strong fixation but requires an accessory incision and neurovascular precautions; outside-in is reproducible and cost-efficient for anterior horn tears; and all-inside reduces soft-tissue dissection and operative time, improving access to posterior/body regions.
- No single meniscal repair technique demonstrates clear superiority in healing or failure rates; outcomes depend on tear characteristics and patient factors. Although modern all-suture all-inside devices show favorable biomechanics, their higher cost and unclear cost-effectiveness limit definitive clinical advantage.
- Technical pearls include optimized portal placement, peripheral bites, protection of neurovascular structures, and avoidance of soft-tissue bridges. Concomitant ACL reconstruction enhances healing – likely via improved stability and marrow-derived biologic augmentation.
- Region-specific guidance is provided using medial and lateral anatomical zoning, aligning suture patterns (inside-out, outside-in, and all-inside) to tear location to preserve biomechanics and reduce complications.

## Introduction

The meniscus is a fibrocartilaginous structure essential for knee joint protection and load distribution (1, 2). Disruption of its integrity can impair biomechanics and accelerate degenerative changes, including cartilage deterioration and osteoarthritis development (3). Surgical management has evolved from meniscectomy – associated with increased joint stress and accelerated degeneration – toward meniscal preservation and repair (4, 5). As an increasing number of meniscal tears are now deemed repairable, the focus has shifted toward preservation to maintain joint biomechanics and function. This shift has contributed to a significant rise in meniscal repair procedures over recent years (5). Historically, the inside-out technique was regarded as the gold standard but posed a risk of neurovascular injury (6). The outside-in approach reduced this risk and is particularly suitable for anterior horn tears (7). More recently, the all-inside arthroscopic technique has gained prominence by eliminating the need for accessory incisions through the use of specialized meniscal suture implants (8). The effectiveness of meniscal repair depends on multiple factors, including tear morphology, joint stability, concurrent ACL reconstruction, and patient-specific characteristics (9, 10). This study aims to review contemporary meniscal repair techniques, emphasizing their relative advantages, limitations, and indications across meniscal regions.

## Surgical technique

### Inside-out

The inside-out meniscal repair technique, first described by Henning *et al.*, involves passing sutures from the intra-articular surface through the meniscus and out of the joint capsule, where knots are tied externally. This method is considered the historical gold standard for meniscal repair, offering strong fixation but requiring an accessory incision to protect neurovascular structures. Henning *et al*. later introduced the concept of an exogenous fibrin clot, demonstrating its potential to enhance meniscal healing (11). This observation laid the groundwork for contemporary biological augmentation strategies, including intercondylar notch trephination, platelet-rich plasma, marrow venting or microfracture, bone marrow aspirate concentrate, stem cell-based therapies, growth factor delivery, and biological scaffolds.

**Figure 1:**
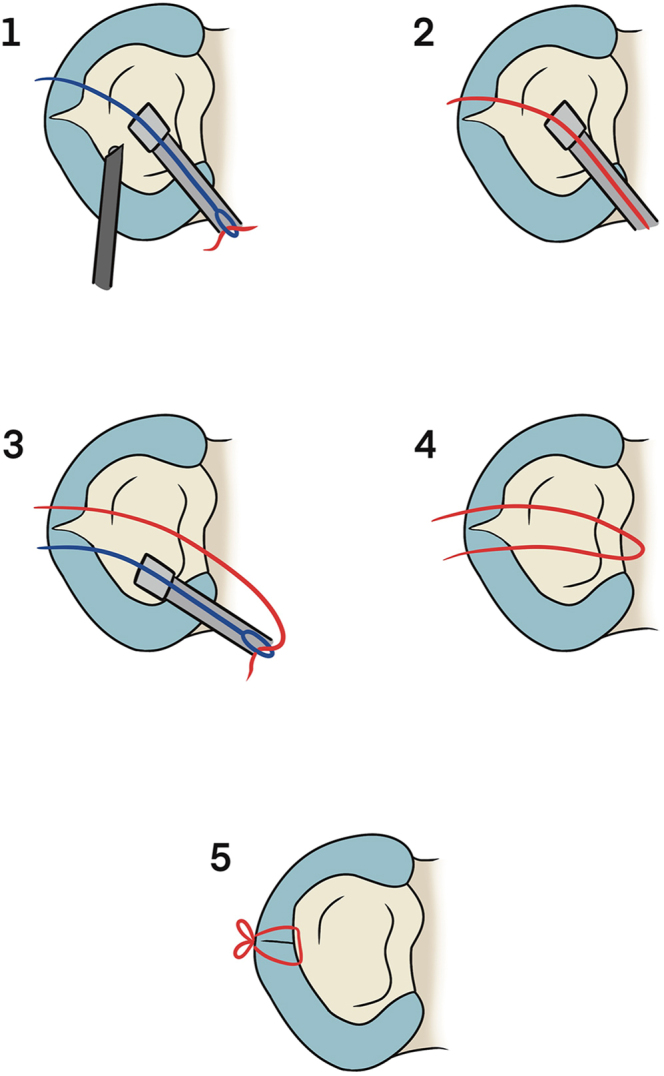
Illustration of the inside-out meniscal suturing technique.

The fundamentals of meniscal repair include accurate tear reduction, stable compression, and an appropriate mechanical environment for healing. Proper lower limb alignment is crucial, as malalignment – particularly varus alignment – results in increased compartmental loading and may compromise meniscal healing. Inside-out sutures support precise reduction and compression of the tear (12). Despite the growing popularity of the all-inside technique, the inside-out technique is still used, and its modifications have been successfully introduced (13). The inside-out technique can be used to treat a range of meniscal injuries, from simple tears to more complex ones, such as radial and bucket handle injuries (14). The main steps of the inside-out repair technique are illustrated in Fig. 1.

### Advantages

Sutures are placed utilizing small-caliber, solid-core needles, which have a reduced diameter compared with the larger-bore needles and implants commonly employed in standard all-inside devices. This supports the preservation of meniscal structural integrity and reduces the likelihood of tear progression (15, 16). Moreover, securing multiple fixation points on the upper and lower surfaces of the meniscus results in stronger suture arrangements, offering better anatomical alignment and greater resistance to biomechanical failure compared with all-inside techniques (17). Additional advantages include easier maneuverability than rigid implants, the possibility of intraoperative adjustment, the use of needles with different curvatures to access challenging tear sites, and the flexibility to choose from various suture materials, such as PDS or non-absorbable threads. Furthermore, this method avoids placing knots inside the joint, which reduces the risk of implant migration and subsequent cartilage damage (18, 19). Traditional suturing techniques are often more cost-effective than all-inside implants, which utilize specialized and typically more expensive technology (20).

### Disadvantages

This technique’s drawbacks arise from requiring an open incision and the dissection of soft tissues outside the joint, which may result in a longer surgery time (21). As a result, the inside-out method tends to be more complex and time-consuming, frequently necessitating a surgical assistant to assist with needle retrieval (22). When employing the inside-out technique, it is crucial to take extra precautions to prevent damage to neurovascular structures (23). Modifying arthroscopic portal placement in the inside-out technique – particularly using anteromedial, accessory anteromedial, or anterolateral portals – can markedly reduce the risk of peroneal nerve injury by optimizing needle trajectory. However, certain combinations, such as middle- or posterior-curved needles used through an accessory anterolateral portal, still pose measurable risk and warrant careful intraoperative consideration (6, 24). In the medial approach, it is important to avoid the saphenous nerve located posteriorly. For the lateral approach, dissection should stay anterior to the biceps femoris tendon to prevent damage to the common peroneal nerve. In addition, during suture retrieval, a broad retractor should be used to safeguard the popliteal artery and its branches from exiting needles (25).

Step-by-step surgical technique

- The camera is introduced via one arthroscopic portal, while the suture device is advanced through the second. Various devices are available for needle insertion.
- The needle directs the thread laterally or medially through the meniscus, ensuring that the thread traverses its full thickness from the inner to the outer surface.
- The needle subsequently penetrates the meniscus at a second location, positioned in close proximity to the initial entry point.
- The thread is passed through the second puncture in the meniscus, ensuring precise alignment. This process establishes a stable seam to support tissue integrity.
- A knot is then tied over the joint capsule within the subcutaneous tissue to secure the suture.

### Tips and tricks

To reduce the risk of injuring the saphenous vein and nerve, the suture technique proposed by Deepak *et al.* can be used, even for posterior horn tears, thereby avoiding a medial incision for medial meniscus repair (26). The disadvantages of this technique are the requirement for special, thin, fluid-venting barrels in the instrumentation and the fact that it can only be used in cases of simultaneous ACL reconstruction (ACLR).

## Tips and tricks from the authors

- Manual bending of the needle delivery system facilitates access to difficult tear sites.
- Check suture tension and meniscal bite before final fixation to ensure stability.
- Extract all sutures from the subcutaneous tissue, avoiding soft-tissue bridges.
- Use sliding knots (SMC, Duncan, and Weston) for reliable compression and easier knot tying.
- Tie knots directly on the capsule, confirmed by visualization or finger palpation, to prevent intra-articular migration and soft-tissue bridging.

### Outside-in

**Figure 2:**
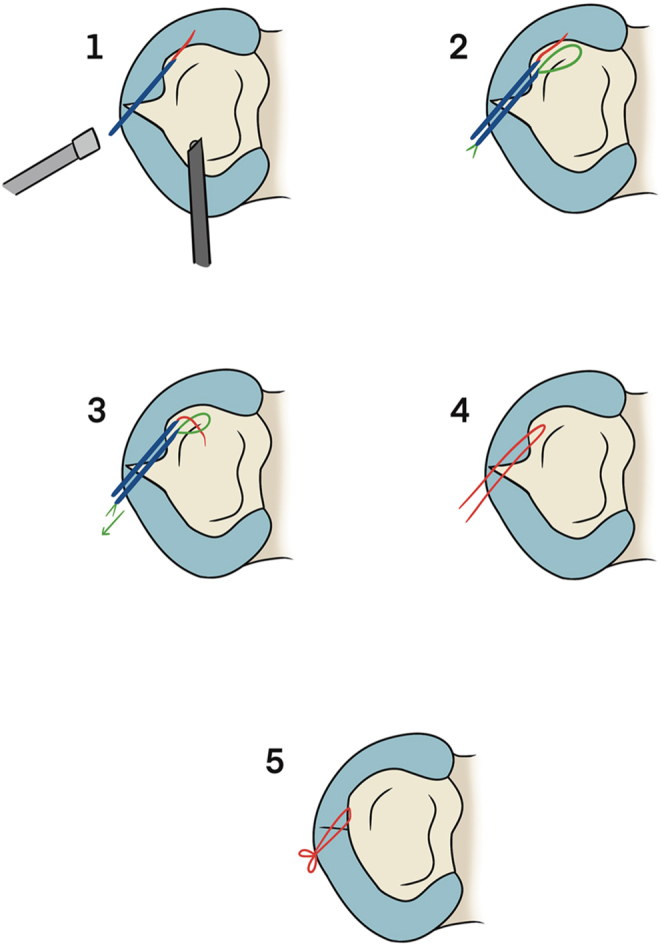
Illustration of the outside-in meniscal suturing technique.

The outside-in approach was initially introduced by Warren *et al.* as a method to reduce the likelihood of peroneal nerve damage (27). The primary indication is an anterior horn tear, as this region is challenging to access with the inside-out and all-inside technique (28). In the following years, this method developed into a widely used approach for repairing tears in both the medial and lateral meniscus (29). The technique and its uses have been updated from Warren’s original mulberry-knot method to include different suture repair patterns, similar to inside-out techniques. The outside-in repair technique is presented in Fig. 2.

### Advantages

Advantages include a minimally invasive surgical approach, high reproducibility of the technique, and low cost due to the use of materials commonly available in operating rooms (30). The healing rate associated with this method is relatively high, with values reported in the literature ranging from 50 to 91%, representing favorable treatment outcomes (31). The outside-in technique is considered the most convenient method for treating meniscal tears of the anterior horn and is also effective for managing tears of the meniscal body. An important consideration is that the knots are secured on the external surface of the joint capsule, which represents a significant advantage (32). Compared with other techniques, this method is associated with a significantly lower incidence of complications, such as neurovascular injury and postoperative joint stiffness (7). The outside-in technique may be particularly advantageous in the small knee joints of young children when stabilizing symptomatic Wrisberg-type discoid menisci, as the inside-out technique requires the use of cannulas that may pose a risk to the articular cartilage (7).

### Disadvantages

This suturing technique is considerably more convenient for repairing the anterior horn and body of the meniscus; however, when addressing the posterior horn, there is a risk of injury to structures within the popliteal fossa. Van Trommel and colleagues reported a lower healing rate on second-look arthroscopy for posterior third meniscal tears treated with the outside-in technique, compared with tears located in the anterior two-thirds (33). In addition, there is a risk of iatrogenic chondral and meniscal injury resulting from penetration by the suture device (7). It is also worth noting that injury to the lateral collateral ligament may occur during the surgical approach (28). The outside-in suturing technique is considered less precise than the inside-out and all-inside methods, as it does not allow for accurate prediction of the needle’s entry point into the meniscus; however, in the hands of an experienced surgeon, the final result becomes more predictable (28).

Step-by-step surgical technique

- Using a single arthroscopic portal, the camera is inserted, and a spinal needle loaded with suture is percutaneously advanced across the meniscal tear.
- A second spinal needle containing a suture loop is then inserted percutaneously.
- The free suture end from the first needle is passed through the loop and retrieved by withdrawing the looped needle.
- The suture is gently pulled to shuttle the free end through the loop and across the tear.
- A knot is tied within the subcutaneous tissue to secure the repair.

### Tips and tricks

Standard needles may be insufficient in length for certain repairs; in such cases, the use of a spinal needle is recommended. Its extended length enables the execution of even technically demanding outside-in sutures (34).

**Figure 3:**
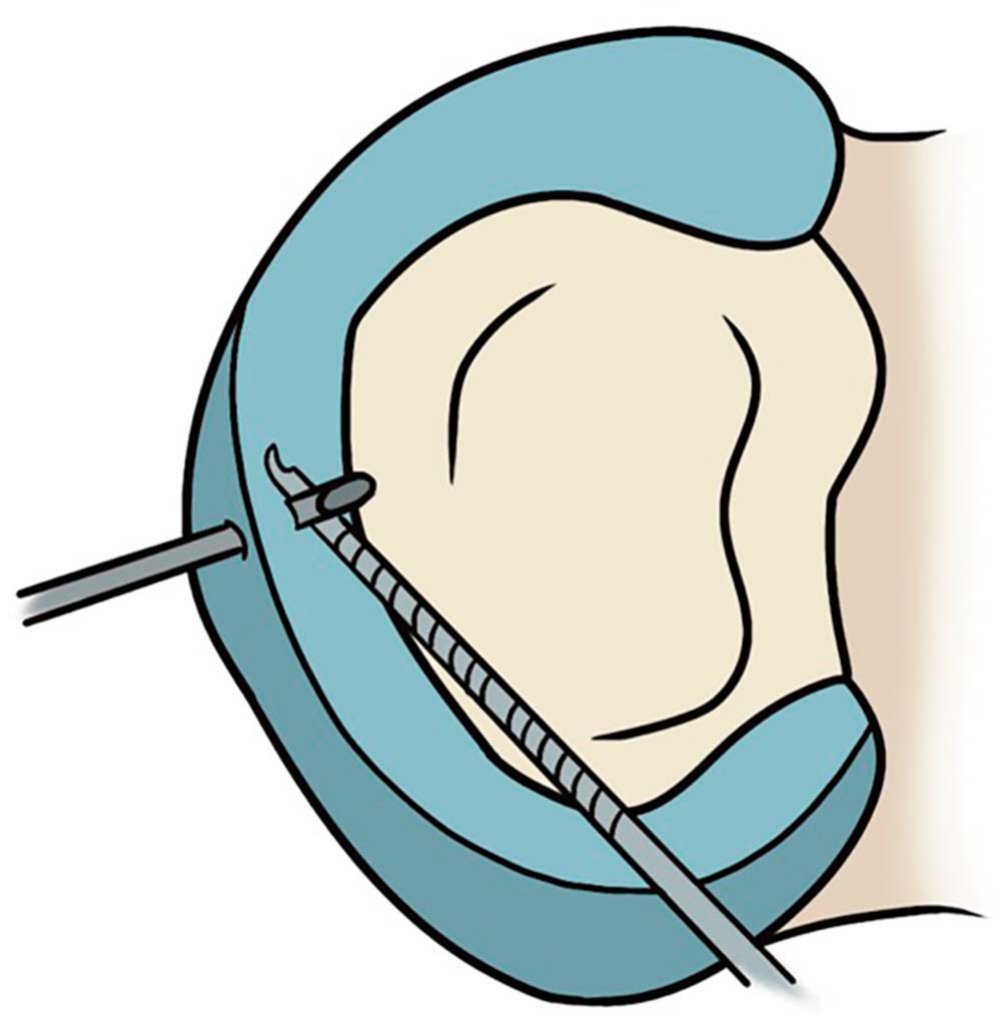
Illustration of needle insertion guided by an arthroscopic hook.

An arthroscopic hook can be utilized to guide needle insertion, while simultaneously aiding in tear reduction and controlled manipulation of the meniscus during tissue penetration (35). Needle guidance using an arthroscopic hook is illustrated in Fig. 3.

## Tips and tricks from the authors

- Provisional stabilization with a percutaneous needle can facilitate suture passage.
- Thread graspers and arthroscopic graspers improve efficiency in thread handling.
- In tight knees, a long loop and passing stitch can be retrieved through the arthroscopic portal and passed outside the joint – avoid soft-tissue bridges.
- If possible, use different-colored threads for the loop and the passing suture to avoid confusion.
- Applying gentle tension on the meniscus with a grasper can ease needle passage.

### All-inside

**Figure 4:**
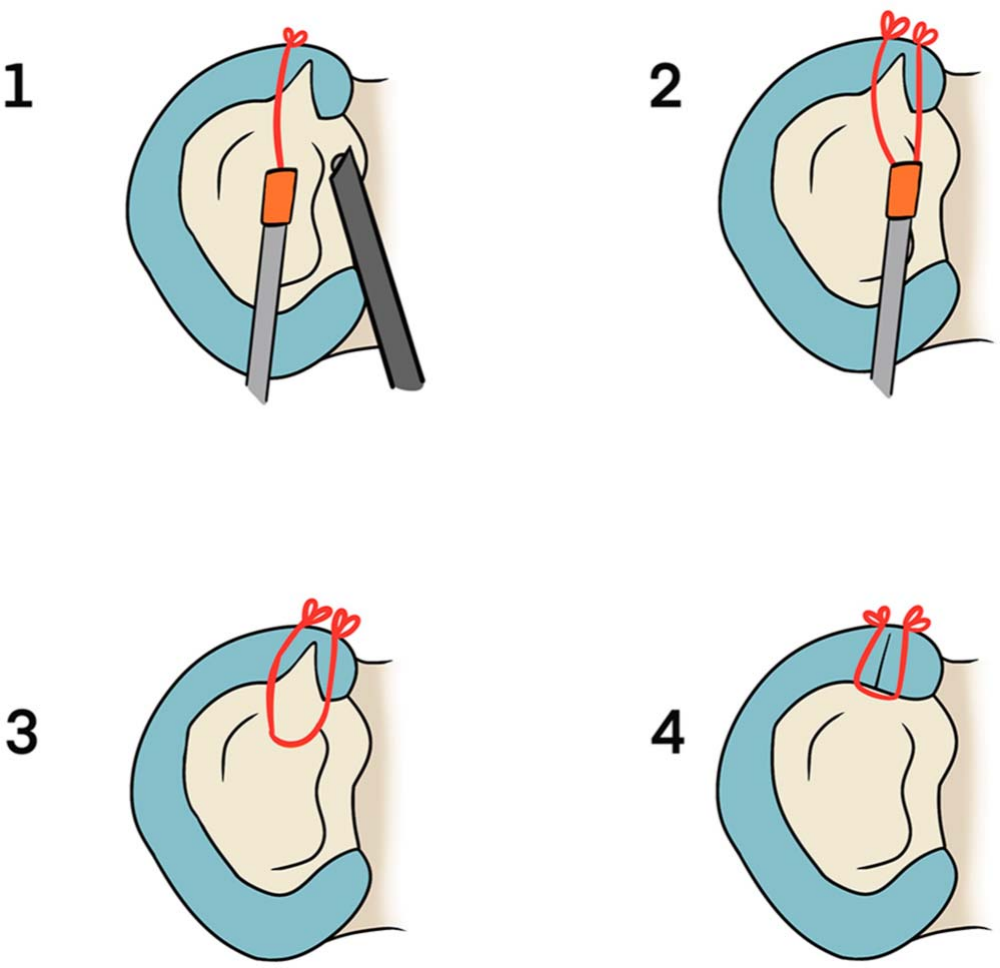
Illustration of the all-inside meniscal suturing technique.

In the 1990s, Morgan and colleagues initiated efforts to identify an optimal method for repairing lateral meniscal horn injuries that would minimize the risk of neurovascular injury to structures within the popliteal fossa (36). The solution proved to be the all-inside suturing technique, which avoids the insertion of instruments into the popliteal fossa (37). One of the major proposed advantages of the all-inside repair technique is its efficiency, as the procedure can be performed more quickly, resulting in shorter operative times and a reduced risk of complications (38). The all-inside repair technique is illustrated in Fig. 4.

### Advantages

An important advantage of the all-inside suturing technique is its speed of deployment. Recent biomechanical and clinical studies suggest that, under certain conditions, all-inside repair may achieve efficacy comparable to – or even exceed – that of other techniques, while also reducing operative time and soft-tissue disruption (39, 40); however, the most recent analyses have not confirmed these findings, suggesting that the comparative advantage of this technique remains inconclusive (41, 42).

Despite the faster application of sutures, the literature remains inconclusive regarding the superiority of the all-inside technique. While some studies report comparable healing rates to other repair methods (43), others suggest higher failure rates in specific tear types or athletic populations (44).

In addition, some studies have reported higher Lysholm scores with all-inside sutures, which may be attributed to several advantages of this technique, including its ease of placement (45, 46). An undeniable advantage is the wide availability of implants specifically designed for various types of meniscal injuries. First-generation all-inside implants, such as arrows and screws, although initially popular, were soon abandoned due to high failure rates and complication risks, as already noted in earlier reports (47, 48). Since then, all-inside repair has advanced considerably, with modern all-suture devices offering smaller diameters that minimize tissue irritation and reduce chondral damage. New delivery systems allow for curved or bendable trajectories, improving access to posterior or complex tear sites. Recent biomechanical studies confirm superior fixation strength and reduced gap formation of all-suture implants compared with older devices and even inside-out sutures (42, 49).

### Disadvantages

The primary disadvantage of the all-inside suturing technique is its cost, as the implants are significantly more expensive than the instruments used for inside-out and outside-in repairs (50). This type of suture is particularly well suited for repairing the posterior horn and body of the meniscus; however, placing all-inside sutures in the anterior horn region remains challenging (51). It is also reasonable to assume that, due to the greater number of components in all-inside implants compared with the instruments used for other suturing techniques, some studies have shown a higher likelihood of mechanical failure (44).

Step-by-step surgical technique

- Using two arthroscopic portals, the camera is inserted through one and the all-inside device through the other.
- The implant is delivered by advancing the device through the meniscus, securing it within the meniscal tissue.
- The device is then passed through a second point in the meniscus, and the suture (implant) is tightened to secure the repair.
- The remaining suture from the implant is trimmed to complete the repair.

### Tips and tricks

Although the use of implants for all-inside suturing is costly, Malinowski *et al.* demonstrated that the all-inside technique can be successfully performed without implants, utilizing only standard instrumentation and non-absorbable PDS sutures (52).

**Figure 5:**
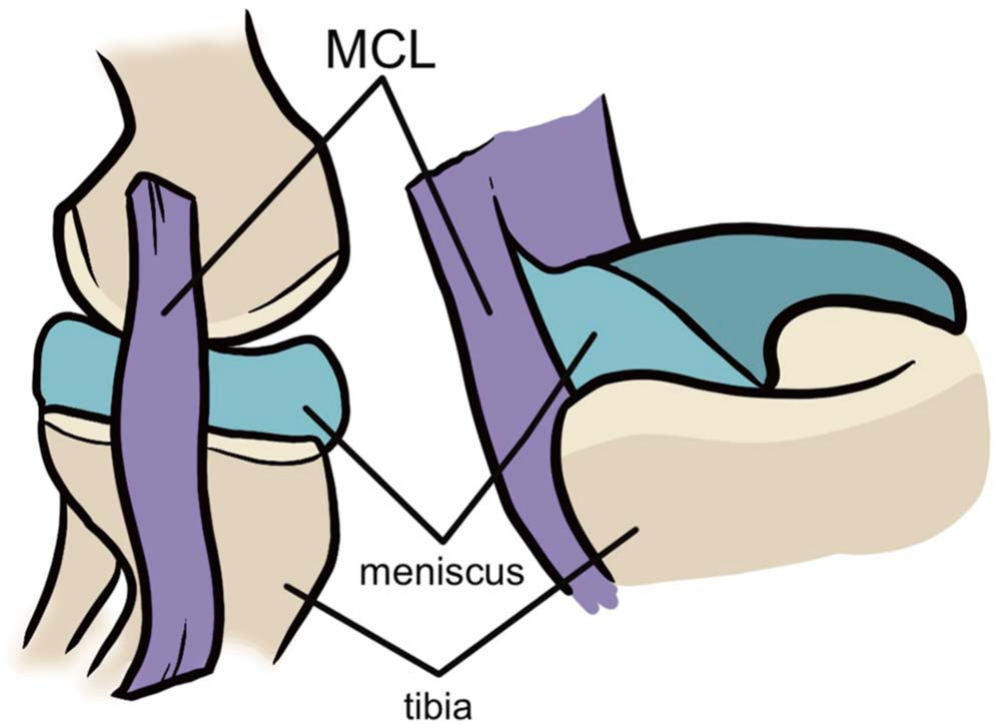
Illustration of meniscal anatomy and the relationship to the MCL.

In this publication, in addition to providing guidelines for the management of horizontal tears in each region of the medial meniscus, the authors emphasize that, to achieve adequate stabilization of medial meniscus tears extending through the majority of the tissue, the placement of sutures at the level of the medial collateral ligament (MCL) is essential. The relationship between the medial meniscus and the MCL is presented in Fig. 5.

**Figure 6:**
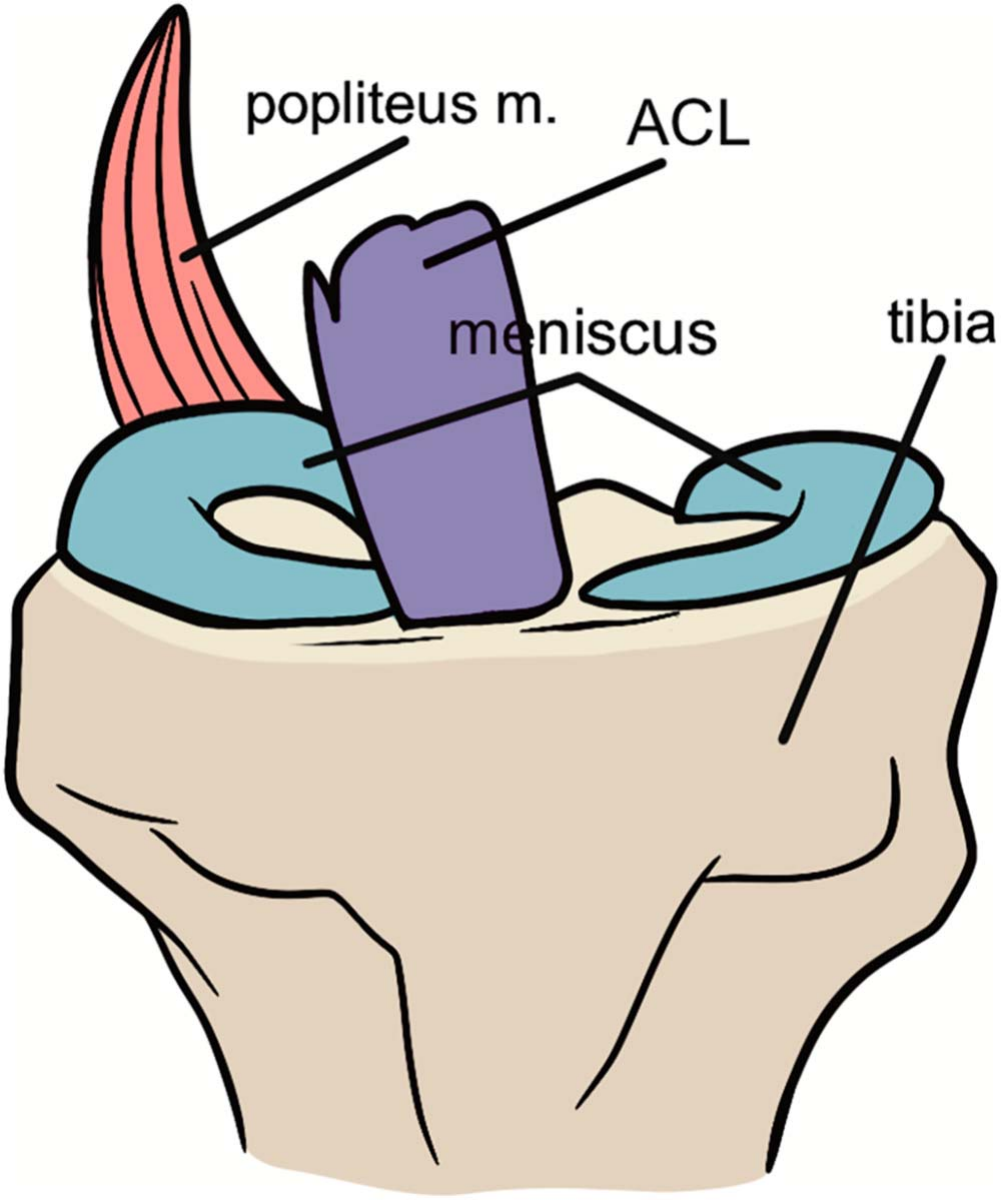
Illustration of meniscal anatomy in relation to the popliteus muscle.

In a separate study, Malinowski demonstrated that lateral meniscal repair can be effectively performed using the all-inside technique without the necessity of costly implantable devices. Given the high mobility of the lateral meniscus, particular caution is required to avoid excessive fixation with sutures. In this study, the authors present a technique for repairing meniscal tears using the all-inside method within the region of the popliteal hiatus, emphasizing the importance of direct visualization to prevent inadvertent suturing of the meniscus to adjacent musculature (53). Meniscal anatomy in relation to the popliteus muscle is illustrated in Fig. 6.

**Figure 7:**
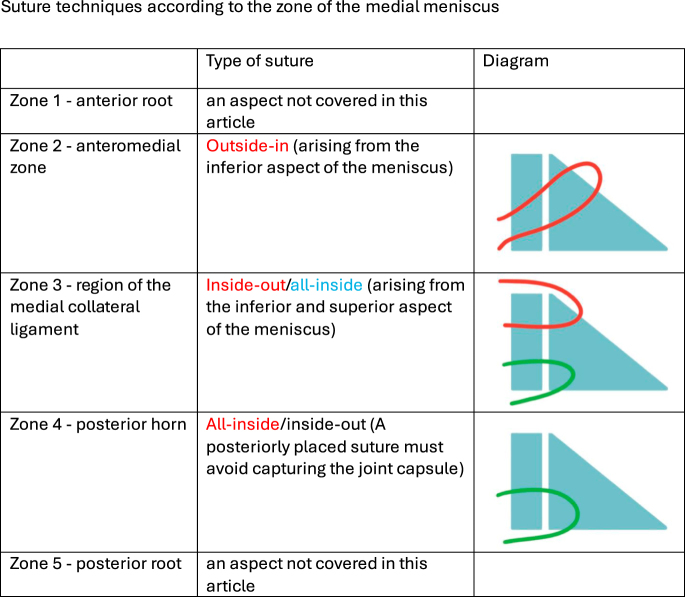
Suture techniques according to the zone of the medial meniscus.

**Figure 8:**
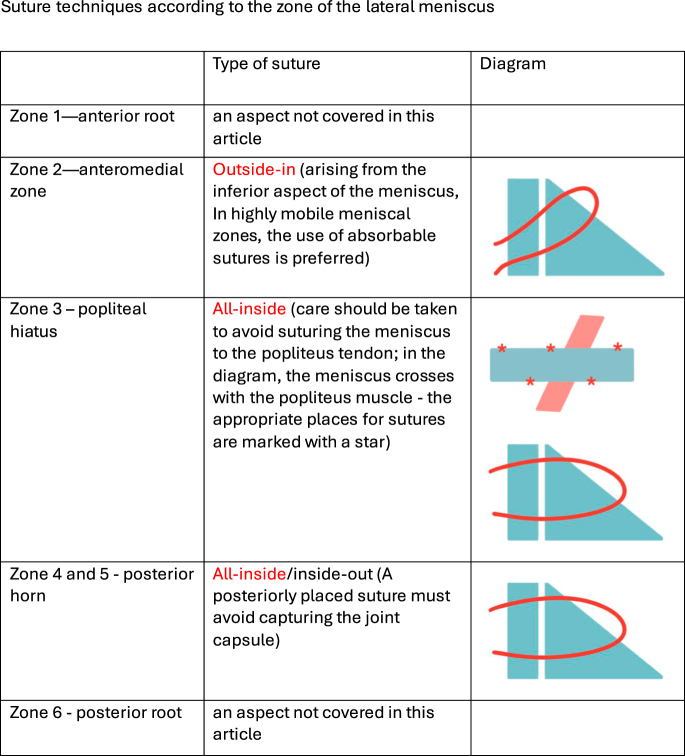
Suture techniques according to the zone of the lateral meniscus.

The depth of the suture device should be limited (around 16 mm) to avoid transcutaneous anchor penetration. Following anchor placement, the posterior aspect of the knee must be inspected to ensure that no skin breach has occurred (54).

## Tips and tricks from the authors

- Use a knot pusher to adjust repair tension.
- Introduce instruments with a ‘half-pipe’ cannula for better joint access.
- A hook introduced from an accessory portal may help guide the device to the correct location.
- Suture the posterior horn from the same portal and the mid-body from the opposite portal for optimal access.
- A central trans-patellar tendon portal can facilitate optimal instrument triangulation and implant trajectory, allowing simultaneous use of two working portals*.*

The advantages and disadvantages of the different meniscal repair techniques are summarized in Table 1.

**Table 1:** Pros and cons of meniscal suturing methods.

	PROS	CONS
Inside-out	Use of smaller-caliber needles (less invasive) Low cost Can be used in every part of the meniscus Very precise technique	Increased risk of neurovascular injury Longer surgical time Requires an open incision Often requires a surgical assistant
Outside-in	Best suited for anterior horn of the meniscus Lower risk of neurovascular injury Materials commonly available Low cost – the cheapest method	Risk of iatrogenic injury to cartilage Reduced precision Potential for lateral collateral ligament injury Risk of injury to popliteal fossa structures when repairing posterior horn tears
All-inside	Fast application – shorter operative times Minimally invasive – reduces the need for additional incisions Wide availability of implants Reduced risk of damage to popliteal fossa structures	High cost Limited access to anterior horn Potential for mechanical failure

According to the classification proposed by Śmigielski *et al.*, the medial meniscus can be divided into five distinct zones, which differ not only in their anatomical characteristics but also in their biomechanical properties (55) (Fig. 7). The lateral meniscus was similarly divided into anatomical zones (56) (Fig. 8). Appropriate suture types can be applied to each of these regions to best support the restoration of the meniscus’s biomechanical roles (57). Presented below is an overview of the various suture types and the anatomical zones of the medial and lateral menisci are presented in Figs. 7 and 8.

### Tips in meniscus suture

The table below outlines key technical recommendations and potential pitfalls in meniscal suturing (regarding all types of suturing). It emphasizes optimal portal placement and tailored approaches for the medial and lateral compartments. A structured surgical sequence is provided to enhance visualization and repair efficiency, while safety measures and principles of suture placement are highlighted to minimize complications and promote healing. Key technical pearls and potential pitfalls in meniscal suturing are summarized in Table 2.

**Table 2:** Tips in meniscus suture.

	Tip	Benefit
Portal placement	Position portals high to avoid contact with the infrapatellar fat pad	Improves visualization and reduces tissue interference
Medial compartment	Extend the medial portal horizontally	Enhances access to the posterior horn
	Perform pie-crusting of the superficial MCL in cases of medial tightness	Facilitates instrumentation and minimizes iatrogenic cartilage damage
Lateral compartment	Create an additional central midline portal to repair the posterior horn near the root	Provides direct access to difficult-to-reach areas
	Place the second anchor in the popliteus tendon when necessary	Considered safe and associated with a low failure rate
Safety considerations	Avoid using the anterolateral portal for suturing the posterior horn of the lateral meniscus	Prevents potential injury to vascular structures
Suture placement	Place sutures as peripherally as possible	Avoids perforations in the avascular (white) zone and promotes healing in the vascularized region

## Discussion

Currently, no single suturing technique is universally optimal for all meniscal tears. The choice of repair method depends on the tear’s morphology and its anatomical location within the meniscus. The crucial role of the meniscus in shock absorption, joint stability, and load transmission has led to a shift from meniscectomy toward preservation techniques, particularly when repair is considered viable (58).

The decision to perform meniscal repair is often guided by specific criteria, including tear location in well-vascularized zones (red–red or red–white), as well as the tear’s size and configuration. Longitudinal vertical and bucket-handle tears are generally considered favorable for repair (59).

Beyond the chosen repair technique, anterior cruciate ligament (ACL) integrity plays a significant role in the success of meniscal healing (38). Cannon and Vittori demonstrated that meniscal healing outcomes were significantly improved in patients who underwent simultaneous ACL reconstruction compared with those without ACL repair (60). This benefit appears to be related not only to restored joint stability but also to the release of bone marrow-derived elements, including growth factors and mesenchymal stem cells, during tunnel drilling in ACL reconstruction, which create a more favorable biological environment for meniscal healing (61, 62).

Contemporary meniscal repair commonly involves one of three approaches: the inside-out technique using double-armed needles, the outside-in technique utilizing straight or curved needles, and fourth-generation all-inside devices. Each method achieves repair by applying suture-based tension across the tear to support biological healing (57). The comparative effectiveness of non-absorbable versus absorbable sutures in promoting meniscal healing remains a subject of ongoing debate (63). Wang Wei and colleagues found a statistically significant difference in overall meniscal healing rates, with second-look arthroscopy revealing superior healing outcomes in the absorbable suture group compared with the non-absorbable group (64). In this study, authors suggested that the superior outcomes with absorbable sutures may be related to reduced long-term foreign body presence, improved tissue integration, and avoidance of complications associated with permanent knots, such as cartilage damage or synovial irritation.

Depending on the location and configuration of the meniscal tear, portal placement can be adapted to optimize intra-articular visualization and instrument access. Typically, the arthroscope is introduced through one portal, while a working instrument is inserted through a separate portal. Alternatively, a central viewing portal may be established for the arthroscope, allowing the use of two additional working portals for simultaneous instrumentation within the knee joint (65).

Several meta-analyses have compared all three meniscal repair techniques. Notably, the study by Elmallah *et al.* provides the following conclusions: the operative time is longer with the inside-out technique compared with the all-inside technique; healing rates are comparable between inside-out and all-inside, but outside-in showed superior healing to all-inside; and no significant difference is observed in complication rates between methods (43).

For patients eligible for meniscal repair, the inside-out technique has traditionally been regarded as the gold standard, owing to its versatility and suitability for a wide range of tear patterns, particularly those involving the posterior horn and body of the meniscus (6, 66). Although the inside-out technique carries an increased risk of vascular injury and postoperative pain, clinical outcomes have remained favorable (6). Horibe *et al.* evaluated 120 patients and reported a 94% clinical success rate, with 73% demonstrating complete healing on second-look arthroscopy (67). Similarly, Steenbrugge *et al.* found that 85% of patients achieved excellent or good outcomes at a mean follow-up of 13 years (68). An alternative to the inside-out technique that avoids the risk of neurovascular injury is the outside-in suturing method. Keyhani and colleagues demonstrated its effectiveness, reporting successful outcomes in 92% of their patients (69). Nonetheless, this technique is most suitable for addressing tears located in the anterior segment of the meniscus (28).

The all-inside technique has gained popularity as a modern alternative, offering a reduced surgical time, improved access to the posterior meniscal horns, and a lower risk of complications commonly associated with inside-out and outside-in approaches (47). Petersen *et al.* compared early- and mid-term outcomes of meniscal repair using flexible all-inside implants and conventional inside-out sutures, finding no significant difference between the two techniques (57). However, in their long-term follow-up, both methods demonstrated higher failure rates compared with the cumulative short-term failure rates previously reported by Grant *et al.* (38) and Kang *et al.* (70).

Earlier studies suggested that newer-generation bioabsorbable screws and anchors yield superior outcomes compared with traditional meniscal arrows. For example, Järvelä *et al.* found that bioabsorbable meniscal screws caused significantly less chondral damage than arrows at two years (*P* = 0.008) (71). However, since then, the field has moved away from screw-based all-inside implants for several reasons. Screws tend to be bulkier and carry a greater risk of cartilage injury from protruding or exposed hardware; screw heads may cause mechanical irritation, especially in tight joint spaces. There are also concerns over implant removal or screw degradation issues and less favorable healing in tears distant from the meniscosynovial junction when using screw devices (72, 73). Because of these complications and the development of thinner, all-suture, or tape-based implants that minimize material-to-tissue mismatch and reduce risk of chondral damage, many surgeons now prefer these modern alternatives.

In a randomized controlled trial of 46 patients, Kise *et al.* found that meniscal arrows (Biofix, Bioscience Oy, Finland) were associated with a 3.6-fold higher reoperation risk compared with all-inside anchors (FasT-Fix/FAST-FIX, Smith & Nephew), despite similar functional outcomes at a two-year follow-up (74).

The aforementioned studies support the initial premise that the choice of meniscal repair technique is influenced by multiple factors, and no single method is universally optimal for all clinical scenarios.

Recent biomechanical studies have shown that modern all-inside, all-suture devices (such as FiberStitch and Arthrex) provide significantly greater fixation strength and reduced gap formation compared with PEEK anchors and conventional inside-out sutures (49, 75).

Moreover, these newer-generation implants demonstrate superior outcomes relative to earlier fixation methods, such as meniscal arrows. A meta-analysis of 3,829 patients with a minimum two-year follow-up reported an overall failure rate of 14.8%, which decreased to 8.5% when repair was performed in conjunction with ACL reconstruction, underscoring the durability of all-inside sutures in selected clinical scenarios (76). Furthermore, long-term data indicate that modern all-inside techniques maintain favorable outcomes, with failure rates of approximately 19.5% at ≥5 years and success rates exceeding 80% when combined with ACL reconstruction at ten years (77).

In knees with a tight medial compartment, irrespective of the meniscal suturing technique employed, the pie-crusting technique may be utilized. This maneuver consists of controlled, stepwise perforation of the MCL, allowing temporary ligamentous relaxation, improved visualization, and increased working space within the medial compartment. Ercan *et al.* demonstrated in a cohort of 68 patients that the use of the pie-crusting technique did not result in significant differences in radiographic measurements when comparing preoperative values with those obtained at 12 and 24 months postoperatively between the control group and the group in which the pie-crusting maneuver was performed (78). Similar conclusions were reported by Wang *et al.* in a systematic review, which additionally demonstrated that patients undergoing the pie-crusting technique achieved significantly higher functional outcomes, as reflected by superior Lysholm and IKDC scores at 24 months postoperatively compared with non-pie-crusting controls (*P* = 0.001 and *P* = 0.0006, respectively). Importantly, no complications or adverse events related to the pie-crusting procedure were reported (79).

## Conclusion

Meniscal repair techniques offer distinct advantages and limitations. The choice of method should be guided by tear location, type, and patient-specific factors. While inside-out remains effective for posterior tears, outside-in suits anterior horn lesions and all-inside offers a minimally invasive alternative with a reduced operative time. No single technique is universally ideal; individualized selection is essential for optimal outcomes.

## ICMJE Statement of Interest

The authors declare that there is no conflict of interest that could be perceived as prejudicing the impartiality of the research reported.

## Funding Statement

This research did not receive any specific grant from any funding agency in the public, commercial, or not-for-profit sector.

## Author contribution statement

MK conceived the concept, performed the literature review, and drafted the manuscript. All authors critically revised the article and approved the final version for publication.
